# Association Between Waist-to-Height Ratio, Isolated and Combined Morbidities and C-Reactive Protein in the Elderly: A Clinical-Epidemiological Study

**DOI:** 10.3390/ijerph110909595

**Published:** 2014-09-16

**Authors:** Jousianny Patrício da Silva, Raquel Patrícia Ataíde Lima, Danielle de Carvalho Pereira, Cassia Surama de Oliveira Silva, Maria da Conceição Rodrigues Gonçalves, Malaquias Batista Filho, Rosália Gouveia Filizola, Ronei Marcos de Moraes, Luiza Sonia Rios Asciutti, Maria José de Carvalho Costa

**Affiliations:** 1 Graduate Program in Nutritional Sciences, Center for Health Sciences/NIESN—Interdisciplinary Studies in Health and Nutrition, Federal University of Paraíba, Castelo Branco, João Pessoa, PB 58059-900, Brazil; E-Mails: raquelpatriciaal@hotmail.com (R.P.A.L); danicarvalhop@hotmail.com (D.C.P.); suramajpa@hotmail.com (C.S.O.S.); 2 Undergraduate Program in Nutrition, Center for Health Sciences, Federal University of Paraiba, João Pessoa, PB 58059-900, Brazil; E-Mails: raulceica@ig.com.br (M.C.R.G.); rosaliafilizola@gmail.com (R.G.F.); mjc.costa@terra.com.br (M.J.C.C.); 3 Undergraduate Program in Mother and Child Health (IMIP), Institute Mother and Child Professor Fernando Figueira, Board of Research, Boa Vista, Recife, PE 50070-550, Brazil; E-Mail: malaquias.imip@gmail.com; 4 Graduate Program in Decision Models and Health, Department of Statistics, Center of Exact and Natural Sciences, Federal University of Paraíba, João Pessoa, PB 58051-000, Brazil; E-Mail: ronei@de.ufpb.br; 5 Department of Nutrition, Faculty of Medical Sciences, João Pessoa, João Pessoa, PB 58010-000, Brazil; E-Mail: luiza.asciutti@terra.com.br

**Keywords:** waist-to-height ratio, C-reactive protein, morbidities, elderly

## Abstract

The aim of this study was to analyze the association between waist-to-height ratio (WHtR) and C-reactive protein (CRP) in the elderly (considering their most prevalent morbidities and lifestyles), to investigate the relationship between this anthropometric index and the presence of the most prevalent morbidities (isolated or combined), and to identify which morbidities (analyzed individually) would have greater associations with WHtR. This cross-sectional population-based epidemiological study of a stratified sampling comprised 170 elderly individuals between 60 and 90 years of age (both genders). Home visits were used to administer questionnaires and to perform anthropometric measurements and blood collection. The mean patient age was younger than 70 years, with women comprising the majority (69.41%) and with 90% of the patients presenting with inadequate WHtR. Hypertension was the most prevalent morbidity in this cohort (58.52%), and when analyzed in combination, hypertension plus obesity were the most frequently diagnosed morbidities (17.65%). Obesity, which was among the most prevalent comorbidities, was the only comorbidity combined with WHtR (*p* = 0.0019). Individuals with no morbidities had lower mean WHtR values compared with individuals with at least one morbidity (*p* = 0.0075). In the multiple linear regression model, it was identified that when individuals had one or more of the most prevalent comorbidities, the mean WHtR increased by 0.0415 (*p* = 0.0065). A correlation between WHtR and CRP (*p* = 0.0379) was also verified. Based on the relationships observed between WHtR (isolated or in combination, data unpublished) and CRP among the elderly, WHtR may represent a screening tool because it is a simple and effective anthropometric index.

## 1. Introduction

As the population ages, increased longevity results in greater numbers of elderly individuals with higher survival rates [1,2,3]. These circumstances represent an interesting field for the development of modern pathophysiological and epidemiological studies of the different contexts in which the process of epidemiological transition is occurring [4,5], thus highlighting the importance of studying the elderly population. Thus, there is concern about regarding the increased incidence of excessive weight or obesity in the elderly [6] due to the correlation of these morbidities with an increased risk for developing non-communicable chronic diseases (NCDs).

As an independent risk factor, abdominal obesity is directly associated with several morbidities [7], as well as an increased risk for cardiovascular diseases, which is the major cause of mortality among NCDs, also in elderly [8] and leads to a widespread use of resources for evaluating abdominal fat accumulation [9,10]. Among the measurements of central obesity, waist-to-height ratio (WHtR) is a particularly excellent tool to discriminate the chances likelihood of obesity related to cardiovascular risks compared with the body mass index (BMI) for both sexes [11]. WHtR is also an excellent tool for discriminating other morbidities, such as diabetes mellitus, atherogenic dyslipidemia and metabolic syndrome, when analyzed individually [12].

Like anthropometric parameters, other variables are able to detect probable health risks, such as the plasma levels of inflammatory markers, particularly C-reactive protein (CRP), which predicts the development of cardiometabolic diseases [13]. The CRP measurement has also emerged as an important predictor of the incidence of NCDs, particularly obesity [14,15]. In this case, the measurement of inflammatory markers is directly related to fat percentage, whereas pro inflammatory cytokines secreted by the adipose tissue reinforce the hepatic synthesis of CRP, supporting the idea that CRP might be elevated in obese individuals [16,17]. However, this predictive ability is not consensual consistent. In one study, no associations between the presence of metabolic syndrome and high CRP levels were found [18]. 

Considering that non-communicable chronic diseases induce elevation in CRP values, and that the prevalence of these diseases is higher in the elderly, as is the elevation of CRP values [19,20,21], it is appropriate to carry out this study, due to its innovative character, aiming to analyze the association between WHtR and CRP in the elderly, as well as the relationship of this anthropometric index with the presence of the most prevalent morbidities in elderly populations when concomitantly analyzed in isolation and in combination.

## 2. Experimental Section

### 2.1. Study Characterization

This epidemiological study, was developed through Household Population Research, which is linked to the “First Diagnosis and Intervention of Food and Nutritional Status and Most Prevalent Non-communicable Diseases in the Population of João Pessoa, Brazil” (I DISANDNT/JP) project, conducted from July 2008 to January 2010. All subjects gave their informed consent for inclusion before they participated in the study. The study was conducted in accordance with the protocol was approved by the Ethics Committee of Center for Health Sciences (CCS), Federal University of Paraíba (protocol number 0493).

### 2.2. Sampling

A representative sample of the city population was stratified using R software [22] and information was collected from individuals in all age groups by home visits; however, this study only included the elderly population. Considering the variables analyzed, the total study sample comprised 170 elderly patients between 60 and 90 years of age (both genders), with complete data based on the sampling model adopted. More specific information about the sample definition and data collection are provided in a previously published study developed by the DISANDNT/JP [23] team.

### 2.3. Data Collection

After training the team and after the pilot study, activities related to the study objectives were performed. The anthropometric evaluations included weight, height and waist circumference measurements. Each measurement procedure was performed in triplicate and the mean values were used. These measures were taken in the households of individuals participating. Excessive weight was assessed by BMI using the parameters proposed by the WHO, which considers general obesity as a BMI level ≥30 kg/m^2^ [24]. Abdominal obesity was assessed using the WHtR ratio for which the recommendation is that the waist value corresponds to half of the height value in centimeters for men and women [25].

After the patients fasted, qualified and trained nursing professionals collected blood samples in the patients’ homes for hs-CRP analysis. The samples were analyzed at the Clinical Laboratory of the “Lauro Wanderley” University Hospital where quantitative analysis was conducted by the agglutination of latex particles using an hs-CRP kit (BioSystems Reagents and Instruments, Barcelona, Spain). The hs-CRP cut-off points for individuals between 60 and 64 years of age were <8.5 mg/dL for females and <7.9 mg/dL for males; for individuals 65 to 90 years of age, the cut-off values were <6.6 mg/dL for females and <6.8 mg/dL for males [26].

The presence of various diseases is common in the elderly [27]. Morbidities were self-reported in the questionnaire, being defined by a “yes” answer to the question: “Have you ever been told to you by a doctor that you have any morbidity? Which one?” [28]. This age group was evaluated, considering the number of individuals without any morbidities, individuals with only one morbidity (*i.e.*, an isolated morbidity), and the number of individuals with one or more morbidities (*i.e.*, combined morbidities).

Notably, demographic and socioeconomic descriptions of the elderly study population, as well as individual lifestyles, including smoking, alcohol consumption, the use of medications and physical activity, were also part of this study. Alcohol consumption was verified using the Quantitative Food Frequency Questionnaire (QFFQ) as reported by Luna *et al.* [23]. The activity level descriptions followed the recommendations of the American College of Sports Medicine [29], which considers a physically active individual as one who performs at least 150 minutes of physical activity per week.

The number of subjects (n = 260) accounted for 12.81% of the entire population found in selected households (n = 2030). The elderly population originally selected (n = 212) comprised 18% of all subjects (n = 1175) and after drop-outs (n = 46), the study group comprised 170 seniors representing 14% of the selected population (n = 1175), higher than the percentage of the elderly in the municipality, which is 9.12% according to the Institute of Municipal Development and State of Paraiba [1].

### 2.4. Statistical Analysis

Descriptive statistics were initially applied, represented by simple frequency using central tendency and dispersion measures (*i.e.*, median, mean, standard deviation and range). Due to the non-normality of some variables, non-parametric tests were used. Spearman’s correlation test between the WHtR and CRP values was applied to analyze the dependence between these variables. To relate the WHtR and CRP variables with the discrete variables, the Wilcoxon test for the comparison of the means was used. Moreover, to determine whether there was an association between the WHtR and morbidities when analyzed in isolation, Fisher’s exact test was used. Finally, to adjust the WHtR and CRP research variables, the following multiple regression models were used:

WHtR = β0 + β1 × CRP + β2 × morbidities (1 or more) + β3 × smoking + β4 × alcohol + β5 × physical activity + β6 × gender + β7 × age + error, and

CRP = β0 + β1 × WHtR + β2 × morbidities (1 or more) + β3 × smoking + β4 × alcohol + β5 × physical activity + β6 × gender + β7 × age + error

A significance level of 5% was adopted for all statistical tests, and the data analysis was performed using R software [17].

## 3. Results

The sample consisted of 170 individuals with an average age younger than 70 years; the majority of the individuals were female. The mean WHtR value was 0.60 cm, with the majority of individuals presenting inadequate WHtR (>0.50) (Table 1). Regarding the anthropometric data, similar percentages of underweight, normal weight, overweight and obesity were observed between males and females. Of note, the values for overweight and obesity were considered to be relevant to the study population because, according to the WHO [19], more than 60% of individuals are overweight. Additionally, with regard to lifestyle, most individuals were sedentary and used medications (Table 1).

Regarding the prevalence of morbidities when analyzed in isolation (*i.e.*, the number of elderly patients with a single morbidity), hypertension was observed to be the most prevalent (32.35%), followed by obesity (8.23%) and diabetes (3.53%) (data not shown). However, when individuals with a single morbidity were analyzed in combination with individuals with combined comorbidities, the percentage of these pathologies (*i.e.*, hypertension, obesity and diabetes) increased to 58.23%, 30% and 12.35%, respectively. However, when only the combined morbidities were analyzed (*i.e.*, individuals with more than one morbidity), it was observed that the highest prevalence occurred for “hypertension + obesity” (17.65%), followed by “hypertension + diabetes” and “diabetes + obesity”.

**Table 1:** Characteristics of the study population.

Characteristics	Mean	Median	SD	Range	N (%)
Demographic and socioeconomic description					
Gender					
Male					52 (30.59)
Female					118 (69.41)
Age (years)	68.80	67.50	7.28	30.00	
Family income (R$)	2137.89	1200.00	2342.84	13,938.00	
Educational level (years)					
0 years					13 (7.64)
Up to 9 years					99 (58.23)
More than 9 years					55 (32.35)
Not provided					3 (1.78)
**Anthropometric characteristics**					
Weight (kg)	66.61	65.00	13.23	78.20	
Height (m)	1.55	1.55	0.09	0.49	
Waist (cm)	92.92	92.50	11.61	72.30	
BMI (kg/m²) ^∞^	27.51	26.84	4.74	27.27	
Normal weight ^§^	22.84				57 (33.52)
Overweight ^§^	27.32	27.26	1.51	4.85	61 (35.88)
Obesity ^§^	33.21	32.19	3.28	13.45	51 (30.00)
WHtR	0.60	0.59	0.08	0.53	170(100)
**Morbidities**					
None *					49 (28.82)
Hypertension *					103 (58.52)
Diabetes *					26 (14.77)
Overweight ^§ ^					60 (34.10)
Obesity ^§^					51 (28.98)
**Lifestyle**					
User of medications					125 (71.02)
User of anti-inflammatories					15 (8.52)
**Biochemical data**					
C-reactive protein (PCR) (mg/dL)	2.83	1.60	3.20	19.19	

Notes: * self-reported morbidity; **^§^** according to the WHO (1998); N = 170, **^∞^** appropriate to age category.

With regard to CRP, most patient values were within the reference range values. Based on the Spearman’s test, due to the non-normality of the WHtR, CRP, alcohol consumption and age variables, the correlation between WHtR and CRP was significant (*p* = 0.0379, r = 0.16), and it could be inferred that as WHtR increases, CRP also increases (Table 2). 

**Table 2:** Correlation between WHtR, CRP, alcohol consumption and the participants’ ages.

Correlation	r	*p*-value
WHtR – CRP	0.16	0.0379 *
WHtR – Age	0.12	0.1152
CRP – Age	−0.05	0.5126

Notes: Spearman’s correlation analysis; * p < 0.05.

**Figure 1:**
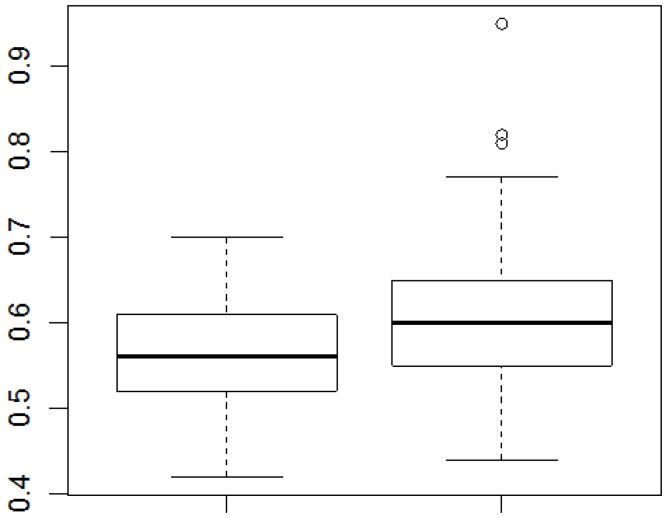
Mean WHtR values between the individuals without morbidities and those with 1 or more morbidities.

When examining whether obesity, hypertension, diabetes, dyslipidemia and cardiovascular diseases (in isolation) were combined with WHtR, the only association was with obesity, with a *p*-value of 0.0019 (data not shown). By linking WHtR and CRP with discrete variables (Wilcoxon test), individuals without comorbidities had lower mean WHtR values compared to individuals with one or more comorbidities (*p* = 0.0075). Moreover, males have had lower WHtR values than females (*p* = 0.0490) (Figure 1).

**Figure 2:**
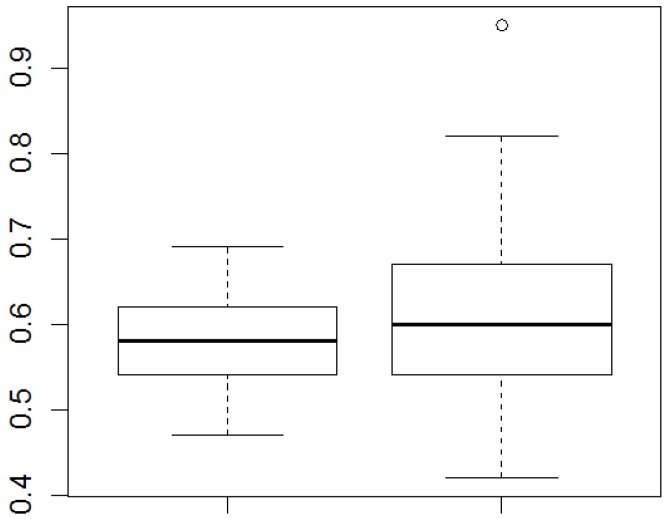
WHtR values for males and females. Wilcoxon test for the comparison of the means; *p* < 0.05.

By investigating the effect of several variables on WHtR in the multiple linear regression model and the subsequent exclusion of non-significant variables, the model of WHtR = 0.5634 + 0.0415 × morbidities (isolated or combined) was obtained, suggesting that in individuals with one or more of the most prevalent comorbidities found in this study, the average WHtR increased by 0.0415 units (*p* = 0.0065). However, in the second model proposed, no variable was significantly combined with CRP (Figure 2).

## 4. Discussion

In the present study, we observed associations between WHtR, isolated and combined morbidity and CRP among the elderly. The association between the WHtR values and the occurrence of morbidities analyzed in isolation (*i.e.*, the number of individuals with only one morbidity) and combined (*i.e.*, the number of individuals with one or more of the most prevalent morbidities) in the elderly population of João Pessoa was analyzed based on a multiple regression model, along with the association between CRP values and WHtR.

The association between high WHtR levels and the occurrence of one or more morbidities (combined) of high prevalence, such as hypertension, obesity and diabetes, is an unpublished result. The average WHtR value of individuals without any morbidity was lower than in individuals with at least one morbidity. A literature search revealed that the associations between anthropometric indicators and morbidities were analyzed only in isolation (*i.e.*, in the number of individuals who were carriers of each of the components related to cardiovascular risk factors, such as type-2 diabetes mellitus or metabolic syndrome) [12,30,31]. 

The high frequency of individuals with high WHtR levels (90%) in this study reinforces the results obtained from a cohort study in Taiwan involving subjects with an average age of 62.1 (±10.9) years, in which the prevalence of high WHtR (>0.50) was 83.9% [31]. Because abdominal obesity plays a key role in mortality due to cardiometabolic diseases, this anthropometric parameter is of great importance as a screening tool in the elderly [12].

With respect to the influence of gender, it has been found that males have lower average WHtR values than females. These data are consistent with the findings of Jayawardana *et al.* [32] who observed that in a sample of 4,485 adults from South Asia over the age of 18 years, men had WHtR values that were significantly lower than those of women. This heterogeneity in the abdominal obesity distribution between genders is also consistent with the results described by Veloso and Silva [33] who found that the prevalence of abdominal obesity among adult women (verified by waist circumference) twice that of men. It is suggested that higher prevalence in women can be attributed to the greater amount of body fat commonly reported for females, pregnancy, hormonal differences and menopause [34]. Regarding these gender differences in the elderly, no justifications were found in the literature, and while it is possible that hormonal differences are an important factor, other factors may also affect fat distribution, including whether adult lifestyles are maintained or change with advancing age.

Regarding the association between WHtR and CRP values in this study, Palacios *et al.* [35] investigated the relationship between adiposity values and cardiometabolic risk factors in adults (21–79 years) living in Puerto Rico and also observed a relationship between these variables, indicating that CRP increases as WHtR increases. Notably, the CRP values observed in this study are mostly within the reference ranges, a fact that may have contributed to the findings relating to the low *p*-value.

The anthropometric parameter WHtR appears to be related to the onset of new cases of cardiovascular diseases in elderly individuals living in the rural area of Taiwan with wherein high WHtR levels (>0.50) were reported [30]. Similarly, Jayawardana *et al.* [32] reported that before the morbidities combined with cardiovascular diseases appear, alterations in the WHtR values had already been observed with the occurrence of diabetes and hypertension when these latter conditions were analyzed in isolation

The relationships found demonstrated in this study reinforce the importance of using this anthropometric indicator as a screening tool because it is related to the most prevalent and most common morbidities analyzed in combination in individuals and not just merely to a certain morbidity in a population, as reported in other studies [10,31]. 

## 5. Conclusions

We concluded that, further studies should be performed with other populations and in different age groups, and cohort studies should be developed to verify whether the improvement of isolated and combined morbidities accompanied by a reduction in the values that are associated with of this relationship, which would better support the recommendation of using WHtR to screen for the combined morbidities that are most prevalent in a given population. It was considered the limitations of study cross-sectional design and self - reported morbidities.
